# Interrelations Between Food Form, Texture, and Matrix Influence Energy Intake and Metabolic Responses

**DOI:** 10.1007/s13668-022-00413-4

**Published:** 2022-03-24

**Authors:** Ciarán G. Forde, Dieuwerke Bolhuis

**Affiliations:** 1 Sensory Science and Eating Behaviour, Division of Human Nutrition and Health, Wageningen University and Research, Wageningen, the Netherlands; 2 Food Quality and Design, Division of Food Technology, Wageningen University and Research, Wageningen, the Netherlands

**Keywords:** Food form, Texture, Matrix, Eating rate, Energy intake, Metabolic response

## Abstract

***Purpose of Review*:**

Nutrition often focuses on food composition, yet differences in food form, texture, and matrix influence energy intake and metabolism. This review outlines how these attributes of food impact oral processing, energy intake, and metabolism.

***Recent Findings*:**

Food form has a well-established impact on intake, where liquids are consumed more than solids and semi-solids. For solids, texture properties like *thickness*, *hardness*, and *lubrication*, and geometrical properties like size and shape influence oral processing, eating rate, and intake. Food matrix integrity can influence nutrient and energy absorption and is strongly influenced by food processing.

***Summary*:**

Food texture and matrix play important roles in modulating energy intake and absorption. Future research needs to consider the often overlooked role of texture and matrix effects on energy and metabolic responses to composite foods and meals. Research is needed to understand how processing impacts macro- and micro-structure of food and its long-term impact on energy balance and health.

## Introduction

Diet-related non-communicable diseases are leading causes of poor health, with the dual epidemic of diabetes and obesity expected to rise globally in the future if current trends continue [1]. Chronic positive energy balance resulting from sustained increased food intake is associated with higher adiposity, prevalence of overweight and obesity, and a greater risk of metabolic syndrome and diet-related chronic disease. When describing the health impact of food consumption, nutrition and dietetic research has traditionally focused on the impact of food composition and its energy density on metabolic health and energy balance. In recent years, researchers have begun to acknowledge the importance of food macrostructural (texture) and micro-structural (matrix) properties in modulating energy intake and metabolic responses to ingested nutrients. This includes the influence of different food forms, food textures, and matrix effects on energy intake and metabolic health.

Food form describes whether nutrients are consumed as solids, semi-solids, or liquids, with well-established preferences and consumption norms for foods that can be consumed as drinks or solid meals. Whereas it is uncommon to drink savory meals, many fruits are often consumed as juices. Excess energy intake from energy-dense liquids has been identified as a risk factor for sustained positive energy balance and weight gain [2]. Consumers eat in response to the cognitive and sensory cues experienced during consumption and whereas the nutrient and energy content of a food is relatively passive in guiding intake within a meal, they exert a strong impact on long-term energy balance [3]. Within semi-solid and solid foods, there are large differences in food texture, which are defined as all of the mechanical, geometrical, and surface attributes of a product that are perceptible by mechanical, tactile, or visual and auditory receptors [4]. The same nutrient load can be consumed as harder or softer textures that differ in their eating rate and intake, though food texture in itself does not directly make a nutrient contribution. Faster eating rates (g/min) and energy intake rates (kcal/min) are a modifiable risk factor for obesity [5], and texture-driven faster eating has been shown to significantly influence energy intake to satiation and metabolic responses for nutrient-matched meals [6]. At a population level, eating at a faster rate is associated with higher daily energy intakes, BMI and adiposity, and increased cardio-metabolic risk [7–9]. The current review summarizes how food form and texture moderate the flow of calories and nutrients through our dietary patterns.

Beyond perceived differences in food texture, we can go deeper to a micro-structural description of a food to look at cell wall integrity and the food matrix, to better understand how cellular structures influence energy and nutrient absorption and metabolism. Whereas a nutrition facts label can describe the gross composition of a product’s macro- and micronutrient and energy content, it does not truly reflect what is absorbed as energy and the true metabolic impact of a food [10••]. These nutrients can be homogeneously dispersed, in a free-form ready for the action of digestive enzymes, or be part of more complex innate food micro-structures that protect or delay their digestion and absorption [11]. For nutrient-matched foods, differences in food matrix may help explain the variability in metabolic responses [12], when food components are orally processed or when nutrients undergo industrial treatments such as grinding, crushing, or thermal processing [13]. If we consider oral processing, digestion and metabolism of nutrients as a physical and chemical “treatment,” then two foods with equivalent nutrient loads but different food textures and matrix properties can also vary in their metabolic responses, in often unseen ways.

This review summarizes current knowledge on the impact of (i) food form, (ii) food texture, and (iii) the food matrix on energy intake and metabolism, and proposes directions for future research.

## Food Form, Eating Rate, and Energy Intake

**Fig. 1:**
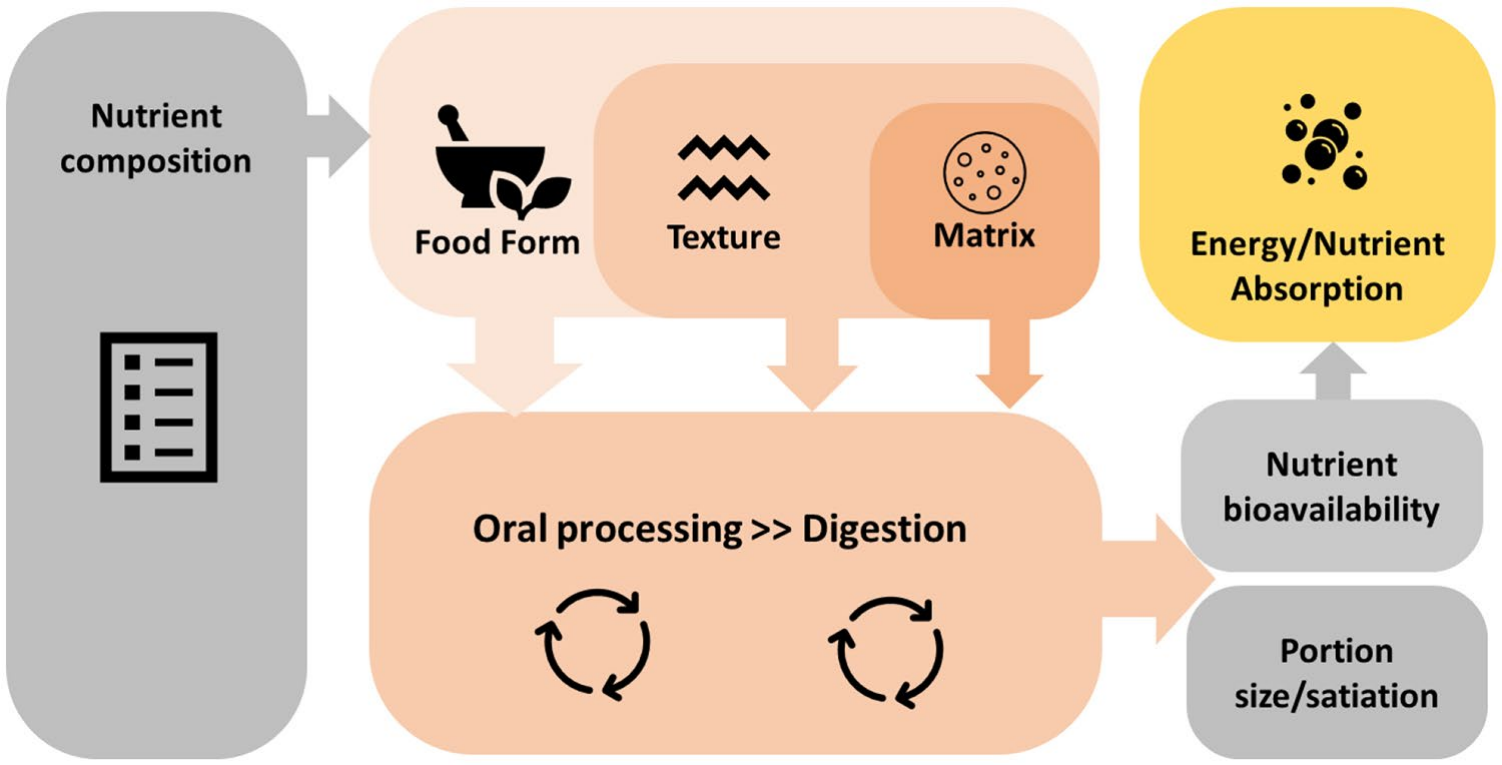
Schematic illustration of the different levels of food structure (food form, texture, and matrix) and the association with energy and nutrient bio-availability

Food structure can be summarized at a macro- and micro-level in terms of its form, food texture, and the underlying structure and integrity of the food matrix, as summarized in Fig. 1. The metabolic consequence of calorie consumption on later appetite is strongly influenced by the form of the food that is consumed. Differences in food form from solids to semi-solids and liquids can influence the portion sizes we consume and the rate and extent of intake within a meal (i.e., satiation), with consensus evidence showing that liquid foods are consumed faster and to a greater extent than semi-solids and solids, respectively [14]. Food form influences both appetitive and nutritional relevant physiological processes important for energy balance [15•].

Liquids can be rapidly consumed with short oro-sensory exposure times and produce a weaker satiety response [16] than the same caloric load consumed as solids [17]. Semi-solids require more movements of the tongue, cheeks, and palate to position the food at the back of the oral cavity and extensive oral processing to reduce the initial structure into smaller particles that can be then lubricated with saliva and agglomerated into a bolus to be swallowed safely [18]. These differences in oral processing influence the rate of consumption and food bolus properties when swallowed. Eating rates for liquids and semi-solids are considerably higher compared to solids (up to 600 g/min vs. 10–120 g/min) [16, 19]. A recent review highlighted that across a large number of studies the consumption of semi-solid compared to liquid versions of the same food reduced eating speed by 20–40% and food intake by 12–34% for the semi-solid versions compared to the liquid versions of foods such as chocolate drinks, custard desserts, and rice porridges (reviewed in [20•]).

Large [21] and small [22] differences in food texture have been shown to influence eating rates, with harder texture being consistently associated with smaller bite size, longer chews per bite, and a longer oro-sensory exposure time [23]. Faster eating rates combined with higher energy density are associated with greater energy intakes [24] and have been shown to influence the onset of satiation and post-meal satiety endocrine responses [25]. When eating rate and oro-sensory exposure were experimentally manipulated for liquids and semi-solid foods, food intake was similar for both suggesting that the duration of oro-sensory exposure is one of the mechanisms by which texture-based difference in eating rate influences food intake [20•]. Eating faster has the dual impact of both increasing energy consumed to satiation and promoting a weaker satiety response, where calorie for calorie comparisons show liquids to have a weaker satiety response compared to semi-solids and solids [26]. Slowing eating rate extends oro-sensory exposure time, which reduces food intake directly by signaling the arrival of calories via the brainstem to higher cortical regions involved in taste and reward [25].

Consumption norms and beliefs regarding expected satiation can also influence the amount of food consumed. Liquid beverages are often consumed to relieve thirst, yet deliver equivalent amounts of energy as snacks or a small meal. When an equivalent amount of energy is consumed as a “beverage” compared to a “snack,” it was shown to affect later appetite responses with the “beverage” condition eliciting a weaker satiety response [27]. Solid and semi-solid foods are often expected to be more satiating than equivalent energy and nutrient load consumed as liquid food [28, 29]. Liquids typically deliver less satiety per calorie consumed, and these learned associations between food form and fullness inform portion selection, where we adjust food intake by selecting larger portions of liquids compared to solids [30].

Combinations of faster eating rates (g/min) and higher energy density (kcal/g) can have a powerful impact on ad libitum calories consumed, with data from a recent randomized controlled feeding trial showing a 50% increase in the energy intake rate (kcal/min) associated with an average > 500 kcal/day increase in energy intake and subsequent weight gain [31]. Energy intake rate (kcal/min) varies widely in the food environment [24] and can directly influence the energy consumed to fullness, with high energy-dense softly textured foods likely to promote passive overconsumption. Diets dominated by foods that have a higher energy intake rate are associated with significantly higher daily energy intake and higher BMI and adiposity [9]. In this regard, changing food form and reducing the energy density of foods create an opportunity to reduce the risk of excessive consumption through a combination of compositional and sensory re-formulation. Understanding the influence of food form on energy intake rate is important as it helps to better identify the dietary patterns linked to excess energy intakes and inform public health guidance to avoid excess consumption of energy-dense liquids, such as sugar-sweetened soft drinks [32].

## Food Texture, Oral Processing, Eating Rate, and Energy Intake

In addition to the large differences between liquids and solid foods, the texture of solid and semi-solid foods has been shown to influence the eating rate and energy intake [33, 34]. Texture properties influence the oro-sensory exposure time, average bite/sip size, and number of chews per bite and through this meal eating rate (g/min) and energy intake rate (kcal/min) during consumption. The “oral breakdown path” offers an explanation of how food breakdown progresses during mastication along three dimensions: degree of structure, degree of lubrication, and time—as described earlier by Hutching and Lillford [35, 36]. In general, foods that need more oral processing are harder or more elastic (degree of structure), have less initial lubrication and require more time to form a swallowable bolus, leading to slower eating rates [18, 20•].

Solid foods are chewed to reduce their size and structure and are fragmented into particles that are lubricated with saliva to bind together in a process known as agglomeration, to form a cohesive bolus that is safe to swallow [36]. We adjust our bite size in response to food structure, taking smaller bites of harder foods that also require more chews per bite to disrupt innate structures, increase surface area, and promote lubrication. Harder foods have been shown to decrease eating rate and food intake by 9–21% across different foods and meals (see Table 1 in [20•]). Both an increased number of chews and longer oro-sensory exposure have been suggested as reasons for the reduction of energy intake when eating harder foods at a slower rate [37, 38]. However, it is important that the difference in perceived “hardness” should be sufficient to observe differences in eating rate and recent data demonstrates that adding fibers to brownies resulted in small changes to the structure that failed to impact oral processing behavior or eating rate. A food’s elasticity is related to its “springiness” or “chewiness” and these parameters relate to how resilient a food is under mastication. Foods that display more elastic behaviors are associated with more chews per bite and a slower rate of eating [23, 39]. Adhesive foods tend to have slower eating rates as they display elastic behavior while adhering to oral surfaces, making it more challenging to agglomerate bolus particles to form a bolus for safe swallowing [40]. For example, within a set of cheese and bell pepper composite foods, changing the texture of the cheese matrix from soft/adhesive to hard/non-adhesive decreased consumption leading to a 7% lower eating rate than cheeses with a hard/non-adhesive matrix, highlighting that eating rate is primarily driven by hardness rather than adhesiveness or stickiness [41].

Foods differ in the amount of saliva required for agglomeration and this depends on both the initial moisture content of the food and its absorption properties [42]. For example, an equivalent amount of bread requires approximately five times more saliva to form a bolus, compared to cooked pasta which is a high water starch gel. Whether knowingly or not, we adapt our oral processing behaviors in response to the specific requirements of a food’s structure and lubrication needs such that low moisture foods, which require more saliva, tend to be chewed for longer to stimulate saliva secretion and incorporation and soften and bind bolus particles [20•]. Many foods are not consumed in isolation and hard/dry foods such as bread and crackers are often consumed with condiments like butter or other types of spreads. This increases lubrication, decreases the number of chews, and thereby speeds up the eating rate [43, 44]. Condiments with low viscosity and high fat have been shown to be most effective in increasing eating rate, but will also increase energy density, to stimulate a higher energy intake rate (kcal/min) and facilitate overconsumption.

The size and the shape of food influence both bite size and the number of chews per gram and small food units are more easily ingested than larger units. Multiple small units can create the impression of “more” than an equivalent amount served as a large unit due to the “numerosity heuristic” [45] where for equivalent amounts an increased number of units creates the impression that the portion is larger [46]. A larger surface area can also increase the consumption of energy-dense condiments and has been shown to influence ad libitum energy intakes [47]. Larger unit sizes can promote a faster eating rate and greater intake compared to smaller unit sizes, for instance when comparing 8-g vs. 32-g pieces of brownies [46], pieces of carrot versus whole carrots [48], and different shapes of vegetables [49]. Foods with a smaller unit size can also require more lubrication than food with larger unit sizes due to the increased surface area available for saliva uptake, and this promotes longer chewing per gram of food and a slower eating rate [12, 22, 50].

Whereas large changes to a food’s hardness may be effective at slowing intake, they can also reduce the sensory appeal and are therefore difficult to implement in a real-world eating context. Smaller changes to a food’s texture have also been shown to increase the oral processing required to manipulate food into a form for safe swallow without a negative impact on sensory appeal. On many eating occasions, we combine various foods together to prepare a meal or snack. The addition of solid food particles in a liquid or semi-solid food can impact oral processing behaviors and energy intake rate (kcal/min) by increasing the need to chew and break down structures, thus prolonging eating time. For example, the addition of peach gel particles to a yogurt decreased the eating rate by 60% while maintaining palatability [51]. Similarly, when smaller and larger particles of granola were added to yogurt in an equivalent weight, the smaller but higher number of granola particles reduced the eating rate and food intake by 5% and 7% respectively, compared to the larger but fewer granola pieces [22]. Adding pieces of bell pepper to cream cheese was shown to decrease eating rate by 9–15% [41], whereas adding apple to yogurt almost doubled oral processing time and decreased eating rate [52]. Taken together, these examples highlight how consumers adapt their oral processing behavior and eating speed in response to the texture challenges they encounter when eating, often in subtle but impactful ways. In this way, food texture influences acute and habitual energy intakes and exerts an influence that is often independent of a food composition and energy density [53]. As such, the form and texture of the food we consume play a functional role in guiding eating behavior and intake and alongside efforts to reformulate foods; texture presents a novel target for sensory and behavioral interventions that aim to increase or decrease food intake within a meal [3, 33].

## Impact of Food Matrix on Energy intake, Satiety, and Metabolic Responses

Nutrition science has traditionally related the health consequences of food consumption to the nutrient and energy content of foods and beverages, and this has been the basis for dietary guidelines for decades [54]. The metabolic and health consequences of food intake assume food composition is the sum of its parts, but does not account for underlying differences in a food matrix structure and subsequent bio-availability of nutrients for digestion and absorption [55]. Food composition only explains part of the dietary variability in our response to ingested nutrients, and extensive research has shown that the same nutrients behave very differently depending on their macro- and micro-structure (Fig. 1). Two foods with identical composition can differ in functionality and have distinct metabolic and physiological impact on consumption [11]. The often overlooked impact of food matrix effects on metabolic responses has been highlighted for a wide range of foods including cereal [56], dairy [57], and fruit products [58]. Without considering these matrix effects, the true health impact of consumption is misrepresented by a food’s nutritional composition alone [59].

Within the dairy product range, processing and matrix structure may enhance interactions between nutrients and modify the metabolic effects of dairy consumption [60, 61]. Differences in micelle structure and composition can influence the digestibility of dairy products and the availability of nutrients for absorption in the large intestine [62]. This is thought to explain some of the discrepancy between a food’s predicted health effect based on nutrient content alone and the reported health effect when consumed as a whole food [63]. Similarly, matrix effects can also moderate the bio-accessibility of many phytochemicals from plant-based foods [64]. For many modern foods, refined fats, carbohydrates, and protein isolates can have different temporal metabolic responses compared to the same ingredients in their natural form [65]. For example, starch bio-availability can be influenced by the degree to which it is refined during processing and classified as rapidly digestible, slowly digestible, and resistant starch depending on the degree to which the initial starch-matrix is maintained and residual matrix interactions with other components such as lipids, proteins, and non-starch polysaccharides [66, 67]. Recent findings have highlighted how carbohydrate texture and matrix interact with an individual’s oral processing behavior and bolus properties during consumption to influence the kinetics of glucose release, with differences ascribed to differences in the underlying food matrix [12, 50, 68]. Similarly, in foods such as nuts, legumes, and cereals, the actual calories absorbed differs considerably from estimates based on their composition, as food matrix structures reduce the digestibility of energy-providing substrates making much of ingested energy inaccessible during normal digestion [10••]. Diets dominated by whole-grain foods that maintain most of their physical integrity during digestion and absorption are therefore likely to be significantly lower in energy intake than estimates based on their food composition alone [69].

Food processing has been implicated in reducing the integrity of indigenous food structures and affecting the rate and extent of post-prandial metabolic responses, when compared to the consumption of whole foods [64, 66]. Concerns have been raised that modern food processing degrades the natural cellular integrity producing “a-cellular” nutrients that can have higher glycemic responses, increased post-prandial lipid responses, and lower satiety [57, 70]. However, evidence for this is equivocal, and processing does not always result in a degraded food matrix and more rapid metabolism and can also be applied to enhance the bio-availability of nutrients that would otherwise not be absorbed (i.e., [71–74]). Processing technologies have been developed to enhance the functional, sensorial, and nutritive attributes of food by modifying their matrix through processes that enhance the release and accessibility of nutrient components through the breakdown of the food matrix. Examples include using an understanding of food matrix effects to enhance the bio-availability of phenolic compounds and bioactive peptides. Research shows that dairy fat when consumed in the form of cheese appears to affect blood lipids differently than when the constituents are eaten in different matrices. Consuming fat within a cheese matrix resulted in significantly lower total cholesterol compared to an equivalent fat intake in a different format [61, 75]. Similarly, reducing a food matrix structural integrity may have a beneficial impact by increasing nutrient bio-availability in food-specific contexts. Processing has also been suggested to disconnect consumers from traditional taste-nutrient relationships with food matrix disruptions and formulation hindering the link between taste quality and intensity and the underlying nutrient content (i.e., sweet taste and mono- and di-saccharide content) [76]. It is currently unclear the extent to which this is true for many modern (re)formulated foods, and further research shows that taste-nutrient relationships are well maintained from low to higher degrees of food processing [77].

Research on almonds shows that despite a high-fat content, lipid metabolism and metabolizable energy are greatly reduced when whole almonds are consumed [78]. Analysis of expectorated bolus samples revealed that the indigenous matrix of the almond cell wall is largely maintained throughout the journey from the oral cavity through the alimentary canal, such that only a low proportion of almond lipids is bio-accessible during digestion. This natural encapsulation of lipids has been proposed as an approach that could potentially enable the structuring of food components within a natural matrix to reduce energy availability and attenuate post-prandial lipid responses [79, 80]. Food processes such as milling, pureeing, extrusion, refining, spray-drying, homogenization, mixing, crushing, roasting, baking, frying, and blanching can decrease the structural integrity of food matrices [10••] and increase the availability of macromolecule components such as fatty acids, amino acids, mono-, and di-saccharides [11]. Processing can reduce the risk of food-borne illness and enhance shelf-life and sensory appeal, but may also influence metabolic responses and absorption. For example, milk is pasteurized to remove pathogenic bacteria, homogenized to subdivide fat globules, and stabilize the lipid layer, which can alter the temporal rates of flavor, protein, and lipid release during consumption and digestion [81, 82]. Similarly, many modern processed foods contain purified or isolated fractions, such as protein isolates, and enzymatically modified ingredients. This has been suggested to increase the biochemical complexity and diversity of nutritional components in the modern diet. Rising concerns about the environmental impact and energy cost of intensive food processing has seen a move to more sustainable and milder processing methods, which are both less resource intensive and less destructive with a focus on producing enriched fractions rather than purified and isolated ingredients. Milder processing methods such as dry processing, or dry fractionation techniques such as “air classification” [83, 84], offer new opportunities to maintain a food’s indigenous matrix with enhanced functionality and nutrient benefits [85]. Further research is needed to compare the metabolic impact of intensive vs. mild processing for different macronutrients and food categories. This will create future opportunities to utilize milder processing and intact food matrices to control, enhance, or moderate the kinetics of nutrient digestion and absorption.

The food matrix poses challenges when estimating the real metabolic impact of food intake on energy and nutrient absorption, but as outlined, may offer new opportunities to tailor food processes and formulations to enhance or reduce the metabolic impact of food consumption [72]. Future research will need to consider how food composition and related matrix effects impact nutrient metabolism, when trying to establish links between diet composition and how it affects health and disease risk. Recent approaches such as the “Food Compass” show potential in going beyond traditional nutrient classification systems to account for a deeper granularity in the description of diet composition across 54 dietary attributes and 9 health-relevant categories, including the degree to which foods have been processed [86].

## Conclusion and Future Directions

The form and texture of food is a powerful “functional” property that guides both the intake behavior and moderates metabolic response to ingested nutrients. Consumers adjust their oral processing behavior and eating rate to the structural challenge posed by the food. The food form, texture, and matrix contribute to individual variability in metabolic responses. Whereas there is a clear understanding of how form and texture influence eating behavior and energy intake [3, 20•], less is known on the complex nature of the food matrix and its influence on nutrient and energy release and absorption. Food processing modifies food texture and matrix where many industrial processes can degrade the food matrix and enhance nutrient bio-availability and digestion [10••, 11]. However, processing can also be applied to slow and reduce the rate of intake, and mild processing may create opportunities to maintain matrix integrity but still enhance the safety and shelf-life [84]. Food processing is a broad term that describes a diverse set of traditional and novel treatments that can have wide-ranging and distinct effects on sensory perception, eating behaviors, and metabolic responses to foods consumed. Processing may degrade the food matrix, but equally, some processes can mitigate the metabolic impact of nutrient intakes, such as the processing of milk into cheese and subsequent buffering of metabolic impact of fat intake in the dairy matrix [55, 60]. Further studies are now needed to explore how micro-structural changes to food matrix integrity can be used to ameliorate post-prandial spikes and support better maintenance of healthy metabolic responses. It currently remains unclear how “mild” processing will impact post-prandial metabolic responses and energy absorption, and future research should quantify metabolic responses across intensive and mild processes for equivalent macronutrient loads.

Future efforts to quantify and communicate a food’s nutritional value should consider nutrient density alongside elements that incorporate the consumption context, eating behavior, and bio-availability of nutrients due to food matrix integrity. Future dietary interventions may consider opportunities to moderate eating rate and intake behavior by changing food texture, while also optimizing nutrient absorption with an enhanced matrix structure. Research has shown that hedonically equivalent food textures can be used to reduce earing rate and energy intake, with estimates suggesting a 20% reduction in eating rate is associated with decreases of 10–14% in ad libitum intake [33]. Future research is now needed to demonstrate the sustained efficacy of these texture-based approaches beyond the current short-term feeding trials.

The current summary highlights opportunities to apply a better understanding of food form, texture, and matrix effects to maintain the sensory appeal of foods, while also moderating potential negative metabolic effects of food consumption. In the future, it will be possible to make recommendations on food texture and matrix design principles that control the speed and extent of consumption, and modulate digestive and metabolic kinetics and absorption of nutrients.
